# Intestinal parasitic infections and associated factors among pregnant women in Ethiopia: a systematic review and meta-analysis

**DOI:** 10.1186/s12884-021-03908-0

**Published:** 2021-07-01

**Authors:** Zelalem Animaw, Addisu Melese, Habtamu Demelash, Girma Seyoum, Abiy Abebe

**Affiliations:** 1 Department of Biomedical Sciences, College of Health Sciences, Debre Tabor University, Debre Tabor, Ethiopia; 2 Department of Medical Microbiology, College of Health Sciences, Bahir Dar University, Bahir Dar, Ethiopia; 3 Department of Public Health, College of Health Sciences, Debre Tabor University, Debre Tabor, Ethiopia; 4 Department of Anatomy, School of Medicine, College of Health Sciences, Addis Ababa University, Addis Ababa, Ethiopia; 5 Traditional and Modern Medicine Research Directorate, Ethiopian Public Health Institute, Addis Ababa, Ethiopia

**Keywords:** Intestinal parasites, pregnant women, systematic review, meta-analysis, pregnancy, Ethiopia

## Abstract

**Background:**

Intestinal parasitic infections (IPIs) are public health problems widely distributed in the world and cause significant morbidity and mortality; many of which occur among women of reproductive age. IPIs caused by helminthes and protozoan parasites are common among pregnant women. Data on the national pooled prevalence of intestinal parasites and associated factors during pregnancy is not documented well in Ethiopia. This review aims at summarizing evidences on the burden of IPIs and associated factors among pregnant women in Ethiopia.

**Methods:**

Published and unpublished studies were thoroughly searched at MEDLINE/PubMed, EMBASE, Google Scholar, CINAHL, Cochrane library and Science Direct. In addition, repositories of Addis Ababa, Gondar and Jimma Universities were searched. Eligible studies were selected following the Preferred Reporting Items for Systematic Reviews and Meta-Analysis (PRISMA) guideline. The pooled prevalence of intestinal parasites and summary odds ratios (ORs) were determined with 95 % confidence intervals (CI). Sub-groups analyses were done based on study region, types of parasites, methods of stool examination and study setting. The statistical analyses were performed using STATA version 14.0 software.

**Results:**

Among 168 retrieved studies, 31 studies with a total population of 12,118 pregnant women were included. The estimated pooled prevalence of IPIs among pregnant women in Ethiopia was 27.32 % (95 % CI: 20.61, 33.87 %). In the subgroup analysis, Oromia and Amhara regions had the highest prevalence with a 29.78 % (95 % CI: 15.97, 43.60) and 29.63 % (95 % CI: 15.37, 43.89); respectively. In addition, studies conducted in the community showed higher prevalence than institution based studies (49.93 % Vs 24.84 %; respectively). The most prevalent type of intestinal parasite identified were *Hookworm* followed by *Ascaris lumbricoides* with a pooled prevalence of 11.2 and 10.34 %, respectively. In our analysis; residence, being bare footed, lack of hand washing habit and eating uncooked/raw vegetables were significantly associated with IPIs among pregnant women in Ethiopia.

**Conclusions:**

Prevalence of IPIs during pregnancy is relatively high in Ethiopia. Poor hygienic practices were identified as risk factors. Based on our finding, targeted preventive measures shall be considered so as to prevent morbidity and mortality due to IPIs.

## Background

IPIs are public health problems widely distributed throughout the world causing significant morbidity, many of which occur among women of reproductive age. Pregnant women are one of high-risk population for these infections [1]. IPIs caused by helminths and protozoan parasites are common among pregnant women and experience more severe infections [2]. However, the severity depends on different factors, including parasitic load, species, inter-pregnancy intervals, nutritional status, poor hygiene and lack of safe drinking water, climate, poverty, immunity status, and the presence of co-existing infections [2–8].

Physiological changes during pregnancy modify the maternal immunity that brings tolerance to the growing fetus and susceptibility to different infections. IPIs are common during pregnancy that aggravates the effect leading to “a double burden to carry” and causes serious problems to the mother as well as to the embryo/fetus [1, 9]. Moreover, IPIs might cause anemia; induce deficiencies of iron, total energy, protein, folate and zinc all of which results in low pregnancy weight gain and intrauterine growth retardation (IUGR), greater risks of infection, low birth weight (LBW) and higher perinatal mortality rates [1, 10–13].

IPIs are reportedly identified as the leading causes of maternal mortality in developing countries especially in the tropics and subtropics [9, 14, 15]. A recent systematic review and Meta-analysis on global prevalence and associated risk factors of IPIs revealed that IPIs in pregnant women is high especially in low and middle income countries [16]. Many other studies were conducted to assess the burden of soil-transmitted helminths (STH) and their effect during pregnancy. Preventive chemotherapy (PC) was introduced as a control program in order to reduce the burden of the infection, but it has been neglected for at-risk women of reproductive ages. The overall coverage of PC was reported as less than 75 % in Ethiopia [17] howing the possibility of the high burden of intestinal parasites. Although it is expected to be high, data on the national pooled prevalence of intestinal parasites and associated factors during pregnancy is not yet documented well in Ethiopia. Therefore; this systematic review and Meta-analysis aims at providing summarized evidence on the burden of IPIs and associated factors among pregnant women in Ethiopia.

## Methods

### Data Bases and Search strategy

**Fig. 1:**
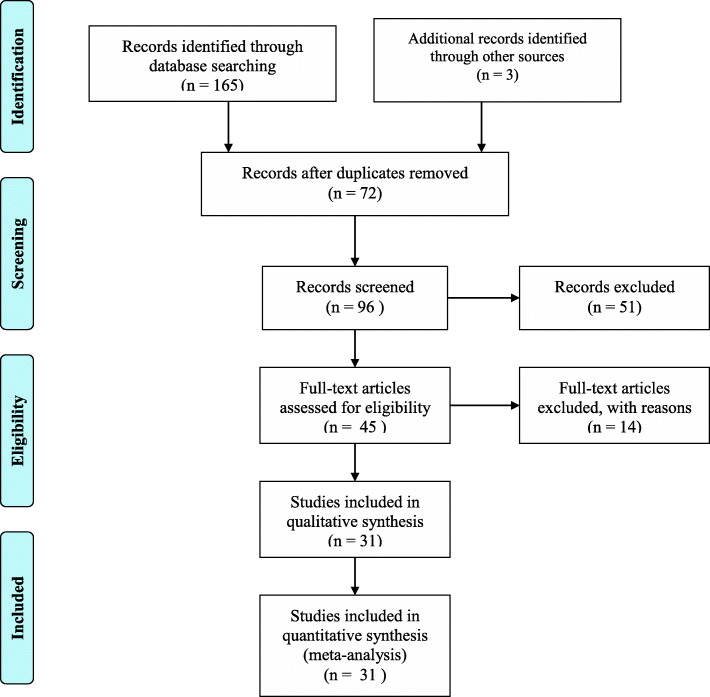
PRISMA chart flow showing article selection process

Both Published and unpublished studies regarding the magnitude of IPIs and associated factors among pregnant women in Ethiopia were thoroughly searched by two authors (ZA and AM) at databases of MEDLINE/PubMed, EMBASE, Google Scholar, CINAHL, Cochrane Library and Science Direct. Additionally, repositories of Addis Ababa University, University of Gondar and Jimma University were searched manually to get unpublished student research works related to the topic. Reference lists of eligible studies were checked to maximize the inclusion of relevant studies. The search was not bounded by year of publication. As a result, all articles published and/or reported up to 25th May, 2020 were included. The Preferred Reporting Items for Systematic Reviews and Meta-Analyses (PRISMA) checklist was strictly followed to select potential studies (Fig. 1).

“Prevalence” “Magnitude”, “Epidemiology”, “Intestinal Parasite”, “Intestinal parasitosis”, “Helmenthiasis”, “*Hookworm” “ Ascaris lumbricoides OR A. lumbericoids”, “Schistosomia Mansoni OR S. mansoni”, “ Giardia lamblia OR G. lamblia”, “ Strongyloides stercolaris OR S. stercolaris”, “Entamoeba histolytica OR E. hisolytica”, “ Trichuris trichiura OR T. trichiura”, “ Hymenolepis nana OR H. nana”, “Taenia species”, “Enterobius vermicularis OR E. vermicularis”*, “Associated Factors”, “Determinants” “Pregnant Women”, “Pregnant Mother” and “Ethiopia” were the key searching terms employed independently and/ or in combination by Boolean operators: “OR” or/and “AND” to bring key concepts together to identify relevant papers. Particularly, studies from MEDLINE/PubMed database were searched by using the following Medical Science Heading (MeSH) terms: (((“parasitic infection“[All Fields] OR “intestinal parasite“[All Fields]) OR (“intestinal diseases, parasitic/epidemiology” [MeSH Terms] OR “intestinal diseases, parasitic“[MeSH Terms])) AND ((((“pregnant women” [All Fields] OR “pregnant mothers“[All Fields]) OR “pregnant mothers attending anc“[All Fields]) AND “pregnant women attending anc“[All Fields]) OR ((“pregnant women“[MeSH Terms] OR “pregnant women/epidemiology“[MeSH Terms]) OR “mothers“[MeSH Terms]))) AND “Ethiopia“[All Fields]. EndNote X7 was also used to manage duplication of articles.

### Inclusion criteria

- Language of publication/written: English.
- Year of publication/report: Up to May 25th of 2020.
- Study design: observational studies (cross-sectional, case-control and cohort).
- Outcome: magnitude of IPIs and/ or associated factors.
- Study population: Pregnant women.
- Study setting: At (institution or community based).
- Study country: Ethiopia.
- Diagnostic modality: Stool examination (either wet mount or concentration or both).
- Type of parasite: Either protozoa or helminthes or both
- Types of articles: Both published and unpublished.
- Types of publication: Peer reviewed, full text articles.

### Exclusion criteria

- Articles which failed to report the number of study participants and number of cases.
- Inaccessible full text articles due to non-responsiveness of the corresponding authors upon frequent inquiry through email by two authors (ZA and AM).
- Systematic reviews and meta-analysis.
- Articles that did not provide calculable prevalence or ORs for associated factors.

### Data extraction

Data pertaining authors' to name with publication year, study period, study design, study setting, study area/region, technique of stool examination, sample size, numbers of pregnant women infected with intestinal parasites and or prevalence of IPIs and species of parasites isolated were extracted from the eligible articles using Microsoft Excel 2013 sheet especially designed for this study. Similarly, a separate data extraction tool was developed for each identified associated factors which contain authors name and publication year. Two by wo tables were also employed to obtain the odds ratio from each study. All associated factors reported by at least two studies were included for pooled analysis.

### Quality assessment

Three authors, (ZA, AM and HD), rated the quality of included studies utilizing Newcastle – Ottawa Scale which enables to assess the quality of each article by their methodological merit, comparability caliber and outcome excellence [18]. In due process, arguments between the authors were settled through in-depth discussion and articles were included upon consensus.

### Statistical analysis

The extracted data were transferred to STATA software version 14.0 to analyze the pooled prevalence of IPIs and odds ratios of the associated factors. Heterogeneity among included studies was assessed using percentage of variance (I^2^) and *P-values*. Random effect model was employed to estimate the pooled prevalence of intestinal parasites and summary odds ratios of factors associated with infections.

Begg’s rank test and Egger’s regression intercept tests were also carried out to indicate the correlation between the effect sizes and sampling variance in order to determine publication bias.

Subgroup analysis was done based on study region, types of parasites (helminthic, protozoa or both species), technique of stool examination (formalin-ether concentration, direct wet mount, or both and others) and study setting (institution or community based).

### Protocols and registration

This systematic review and Meta-analysis is registered on PROSPERO under a registration number of **CRD42020189115** and can be accessed at https://www.crd.york.ac.uk/PROSPERO.

## Results

### Characteristics of the included articles

A total of 31 studies were included in this systematic review and meta-analysis. The number of pregnant women participated in the studies ranged from 85 to 783 [19, 20] constituting a total population of 12,118. The reported prevalence of IPIs was ranging from 7.3 % [21, 22] to 76.0 % [20]. Twenty nine studies [4, 5, 10, 12, 19–43] were cross-sectional while the rest two were case-controls [11, 44]. The studies were published from 2013 up to 2020. Regarding geographical distribution, 10 studies were reported from Amhara region [4, 10, 20, 25, 33, 34, 36–38, 40], 8 from SNNPR [23, 26–29, 42–44], 8 from Oromia [5, 11, 12, 24, 32, 35, 39, 41], 4 from Tigray [19, 22, 30, 31] and One from Addis Ababa [21] (Table 1).

**Table 1:** General characteristic of the included articles for systematic review and meta-analysis pertaining magnitude and associated factors of IPIs among pregnant women in Ethiopia

Author, publication year	Study period	Study design	Study setting	Study area/Region	Technique of stool examination	Sample size	Cases	Prevalence (%)	Parasite species isolated
Feleke and Jember, 2018 [20]	November 2015 to January 2016	cross-sectional	Community based	Mecha, Amhara	formalin-ether Concentration	783	595	76.0	* A. lumbricoides, S. mansoni*, Hookworm, *S. stercolaris*
Bolka and Gebremedhin, 2019 [23]	June and July 2018	cross-sectional	Institution based	Wondo Genet, SNNPR	formalin-ether Concentration	349	135	38.7	Hookworm, *S. mansoni, A. lumbricoides, T. trichiura, G. lamblia, E. histolytica*,
Derso et al., 2016 [4]	November 2013 to January 2014	cross-sectional	Institution based	Felege Hiwot Hospital, Amhara	formalin-ether Concentration	384	121	31.5	Hookworm, *S. mansoni, A. lumbricoides, S. stercolaris, T. trichiura, G. lamblia, E. histolytica, H. nana, Taenia species*
Yesuf et al., 2019 [24]	April 1 to May 15, 2019	cross-sectional	Institution based	Four Health centers in Lalo Kile, Oromia	Both	315	138	43.8	Hookworm, *A. lumbricoides, S. stercolaris, T. trichiura, G. lamblia*
Alem et al., 2013 [25]	February to May 2011	cross-sectional	Institution based	Azezo Health Center. Amhara	formalin-ether Concentration	384	55	14.3	Hookworm, *S. mansoni, A. lumbricoides, G. lamblia, E. histolytica, H. nana, Taenia species*
Lebso et al., 2017 [26]	May-June 2015	cross-sectional	Community based	Lemo, SNNPR	Direct wet mount	504	161	31.9	Hookworm, *T. trichiura, A. lumbricoides, Taenia species*
Kefiyalew et al., 2014 [12]	March to June 2013	cross-sectional	Institution based	Bisidimo Hospital, Oromia	Both	258	96	37.2	Not reported
Bekele et al., 2016 [27]	February 16 to April 8, 2015	cross-sectional	Institution based	Arba Minch hospital, SNNPR	formalin-ether Concentration	332	40	12.0	Hookworm, *A. lumbricoides, G. lamblia, E. histolytica, Taenia species*
Gedefaw et al., 2015 [28]	January to March 2014	cross-sectional	Institution based	Otona Hospital, SNNPR	Direct wet mount	363	69	19.0	Hookworm, *S. mansoni, A. lumbricoides, T. trichiura, G. lamblia, E. histolytica, E. vermicularis*
Asrie, 2017 [10]	January to March 2015	cross-sectional	Institution based	Aymiba health center, Amhara	Direct wet mount	206	16	7.8	Hookworm, *E. vermicularis, A. lumbricoides*,
Getahun et al., 2017 [29]	March 01–April 30 2015	cross-sectional	Institution based	Butajira hospital, SNNPR	Both	217	28	12.9	Not reported
Ejeta et al., 2014 [5]	April to May, 2014	cross-sectional	Institution based	Nekemte Hospital, Oromia	Direct wet mount	286	22	7.7	Not reported
Zekarias et al., 2017 [43]	April 3 to May 3, 2017	cross-sectional	Institution based	Mizan Tepi hospital, SNNPR	Direct wet mount	306	70	22.9	Not reported
Melku et al., 2014 [38]	March 1 to April 30, 2012	cross-sectional	Institution based	Gondar University Hospital Amhara	Direct wet mount	302	80	26.5	Hookworm, *E. histolytica, A. lumbricoides*,
Tesfaye, 2015 [42]	October 1 to 30, 2013	cross-sectional	Institution based	Nigist Eleni hospital, SNNPR	formalin-ether Concentration	258	76	29.5	Hookworm, *T. trichiura, A. lumbricoides, H. nana, Taenia species*
Mengist et al., 2017 [13]	November 2015 and January 2016	cross-sectional	Institution based	Five Health Centers in Wollega, Oromia	Both	372	92	24.7	Hookworm, *S. stercolaris, A. lumbricoides, H. nana, Taenia species*
Getachew et al., 2013 [32]	August to September, 2011	cross-sectional	Community based	Gilge Gibe, Oromia	formalin-ether Concentration	388	162	41.8	Hookworm, *T. trichiura, H. nana, E. vermicularis, A. lumbricoides*,
Tefera. 2014 [41]	April, 1 – June 30, 2014	cross-sectional	Institution based	Sher-Ethiopia hospital, Oromia	Both	748	436	58.3	* A. lumbricoides, T. trichuria*, Hook worm, *S. mansoni*
Kumera et al., 2018 [36]	July to August 2016	cross-sectional	Institution based	Debre Markos Hospital, Amhara	Both	234	64	27.4	Hookworm, *E. histolytica* *A. lumbricoides, G. lamblia*,
Kebede et al., 2018 [22]	April 1–30, 2018	cross-sectional	Institution based	Suhul hospital, Tigray	Not mentioned	480	35	7.3	Not reported
Berhe et al., 2019 [30]	April to September 2018	cross-sectional	Institution based	Adigrat Hospital, Tigray	Direct wet mount	304	54	17.8	*E. histolytica, G. lamblia*,
Helion Belay et al., 2020 [34]	January 11 to February 20, 2017	cross-sectional	Institution based	Health centers in Dembia, Amhara	Not mentioned	685	158	23.1	Not reported
Kenea et al., 2018 [35]	January to July 2016	cross-sectional	Institution based	Mettu, Bedele, and ,Darimu hospitals, Oromia	Not mentioned	416	32	7.7	Not reported
Fassil, 2016 [21]	December 2015 to February 2016	cross-sectional	Institution based	Selam Health Cener, Addis Ababa	Not mentioned	480	35	7.3	Not reported
Shiferaw et al., 2017 [40]	March to June, 2015	cross-sectional	Institution based	Anbesame health center, Amhara	Direct wet mount	180	38	21.1	Hookworm, *S. mansoni*,
Kumera et al., 2018 [37]	January to February 2016	cross-sectional	Institution based	University of Gondar Hospital, Amhara	Both	402	126	31.3	Hookworm, *E. histolytica* *A. lumbricoides, H. nana* *G. lamblia, S. stercoralis, S. mansoni, Tanea species*
Tulu et al., 2019 [11]	September 7 to October 25, 2017	case control	Institution based	Health facilities in Horo Guduru, Oromia	formalin-ether Concentration	573	101	17.6	Hookworm, *T. trichiura, H. nana, A. lumbricoides*
Gebrehiwet et al., 2019 [31]	Not described	cross-sectional	Institution based	Maytsebri Hospital, Tigray	Kato-Katz	448	229	51.1	Hookworm, *T. trichiura, A. lumbricoides*
Hailu et al., 2019 [33]	February to June, 2017	cross-sectional	Institution based	Health centers in W/Gojjam, Amhara	Formol-ether concentration	743	276	37.1	Hookworm, *E. histolytica* *G. lamblia, A. lumbricoides*
Weldekidan et al., 2018 [44]	February 16 to May 8, 2017	case control	Institution based	Health facilities in Durame, SNNPR	formalin-ether Concentration	333	92	25.2	Hookworm, *G. lamblia, A. lumbricoides*
Gebreegziabiher et al., 2014 [19]	October 2011 to July 2012	cross-sectional	Institution based	Mekele, Ayder, Semen Heath center, Tigray	Direct wet mount	85	30	35.3	*T. trichiura, E. histolytica* *A. lumbricoides, H. nana* *G. lamblia, S. mansoni, E. vermicularis*

### Pooled prevalence of intestinal parasitic infection among pregnant women in Ethiopia

**Fig. 2:**
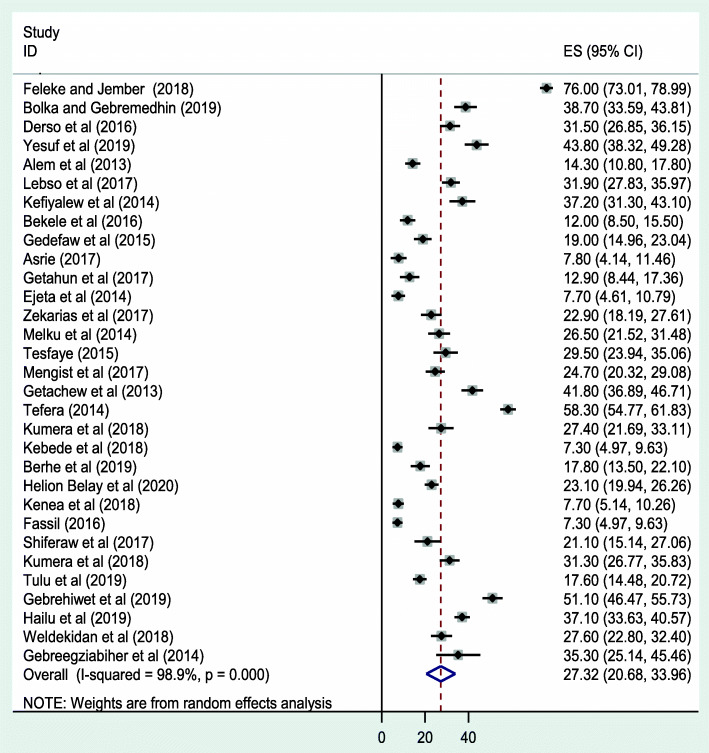
Forest plot of the pooled prevalence of IPIs among pregnant mothers in Ethiopia

The estimated pooled prevalence of IPIs among pregnant women in Ethiopia is 27.32 % (95 % CI: 20.61, 33.87; I^2^ = 98.9 % *p* = < *0.001*) (Fig. 2). Statistically significant heterogeneity was observed in the estimation of this pooled prevalence. Begg’s rank and Egger’s regression tests were carried out to statistically determine publication bias [45]. Based on the results, absence of significant publication bias was declared objectively with *P = 0.068* and *P =* 0.063, consecutively.

### IPIs and anemia

Among the included articles 16 of them [5, 10–13, 21, 22, 25, 26, 28, 33, 35, 37, 38, 41, 44] reported that IPIs are significantly associated with anemia during pregnancy while 4 studies [27, 29, 34, 43] revealed that there is no association between occurrence of anemia among pregnant mothers who contracted IPIs. A single study disclosed that IPIs during pregnancy are significantly associated with maternal under-nutrition [36].

### Types of intestinal parasite

In this study, the most prevalent type of intestinal parasite identified was *Hookworm* followed by *Ascaris lumbricoides* with a prevalence of 11.12 % (95 %CI: 8.21, 14.02) and 10.34 (95 %CI: 7.09, 13.59); respectively. The least reported parasites are *Enterobius vermicularis* and *Taenia species* (Table 2).

**Table 2:** Pattern of Intestinal parasites among pregnant women

Types of parasites	Species of parasite	No of studies	Pooled prevalence (95 % CI)	I^2^	P-values
Helminthic	Hookworm	22	11.2 % (8.21, 14.02)	97.3 %	< 0.001
	*Ascaris lumbricoides*	20	10.34 % (7.09, 13.59)	97.9 %	< 0.001
	*Schistosomia mansoni*	10	3.42 % (1.82, 5.01)	95.3 %	< 0.001
	*Strongyloides stercolaris*	5	1.56 % (0.31, 2.82)	91.7 %	< 0.001
	*Trichuris trichiura*	11	2.85 % (1.76, 3.94)	84.3 %	< 0.001
	*Hymenolepis nana*	8	1.09 % (0.56, 1.63)	50.8 %	0.047
	*Taenia species*	7	0.94 % (0.57, 1.31)	65.4 %	0.022
	*Enterobius vermicularis*	4	0.81 % (0.02, 1.63)	58.31	0.031
Protozoa	*Giardia lamblia*	13	5.25 % (3.41, 7.09)	89.5 %	< 0.001
	*Entamoeba histolytica*	11	6.89 % (4.07, 9.71)	94.0 %	< 0.001
Mixed	Mixed infection	11	7.08 % (4.18, 9.98)	95.6 %	< 0.001

### Subgroup analysis

**Fig. 3:**
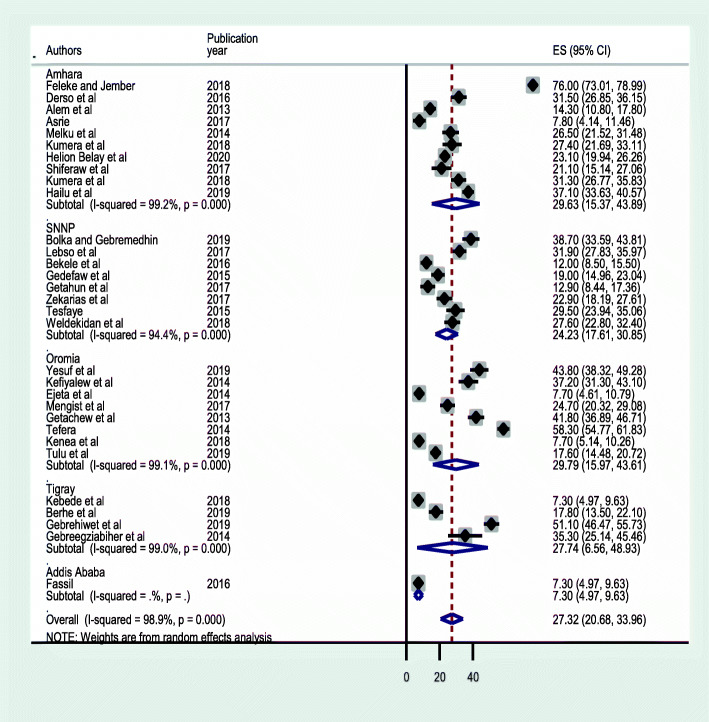
Forest plot of subgroup analysis based on regions

**Fig. 4:**
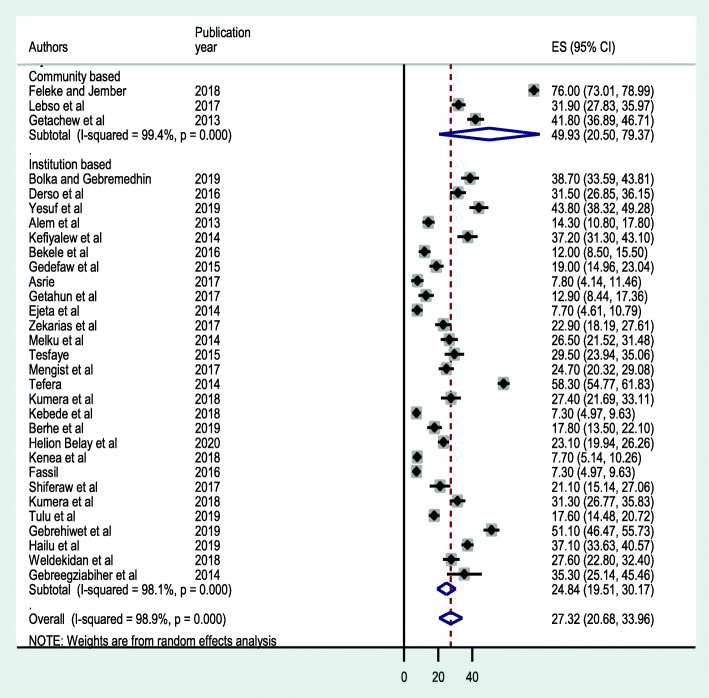
Forest plot of subgroup analysis based on study setting

**Fig. 5:**
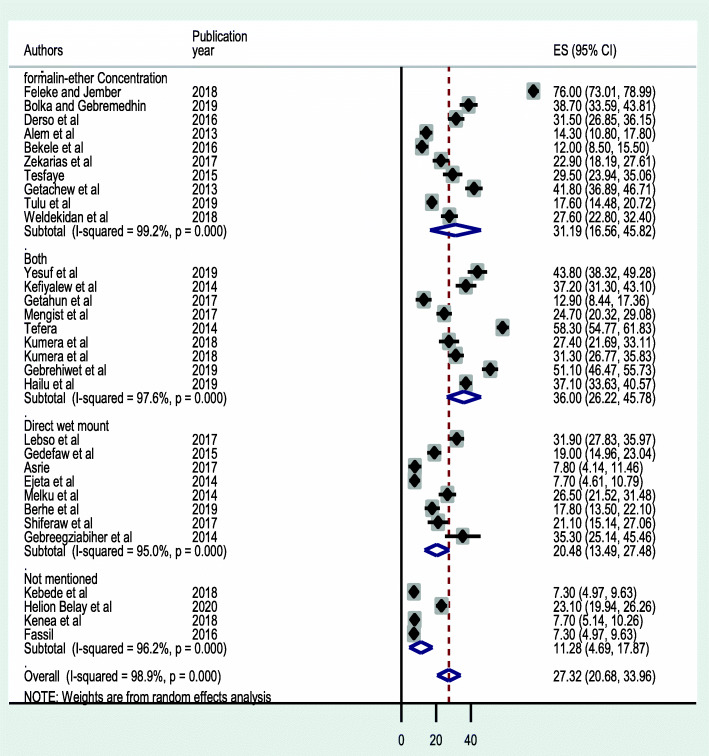
Forest plot of subgroup analysis based on techniques of stool examination

A subgroup analysis based on study regions revealed that Oromia had the highest prevalence estimate accounting 29.78 % (95 % CI: 15.97, 43.60) closely followed by Amhara region 29.63 % (95 % CI: 15.37, 43.89), Tigray region 27.74 % (95 % CI: 6.56, 48.93) and SNNPR 24.23 % (95 % CI: 17.61, 30.85) (Fig. 3). Another subgroup analysis by study setting showed higher prevalence in studies done in the community than studies done institutions with a pooled prevalence of 49.93 % (95 %CI: 20.49, 79.37) and 24.84 % (95 %CI: 19.51, 30.17); respectively (Fig. 4). A similar analysis depending on the technique of stool examination indicates a combination of formalin-ether concentration and direct wet mount reported a higher prevalence of IPIs (35.99 %; 95 % CI: 26.22 ,45.78) than studies that used a single stool examination to diagnose intestinal parasites (Fig. 5).

### Factors associated with intestinal parasitic infection among pregnant women in Ethiopia

**Fig. 6:**
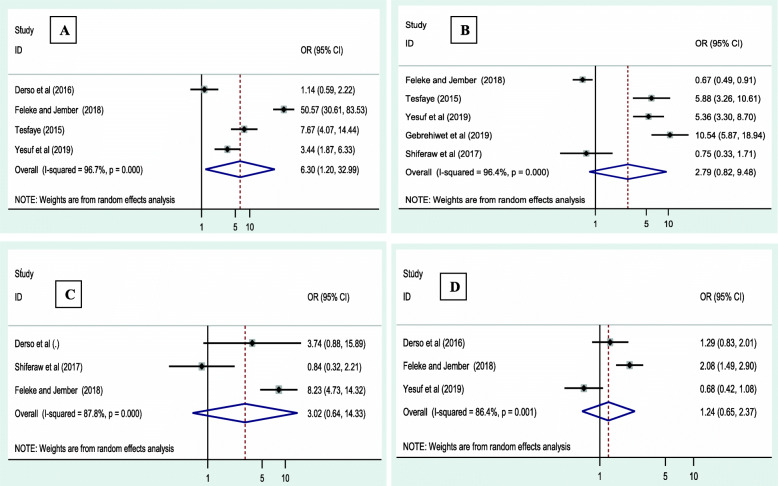
Forest plot indicating odds ratio of factors associated with intestinal parasitic infection among pregnant women in Ethiopia. (**A)** Residence, (**B)** Bare foot, (**C)** Hand washing habit and (**D)** consume raw vegetables.

Our analysis identified that residence area, being bare footed, hand washing habit and eating uncooked/raw vegetables have significant association with the occurrence of IPIs among pregnant women. Pregnant women from rural areas were 6.3 more likely to develop IPIs when compared to urban dweller pregnant mothers (OR = 6.31; 95 % CI: 20, 32.99; *P* = 0.002) (Fig. 6 A). Likewise, a barefooted women were 2.79 times more likely to be infected with IPIs than those who wore shoes (OR = 2.79; 95 % CI: 1.82, 9.48; *P* = 0.01) (Fig. 6B). Similarly; pregnant women who had no hand washing habit and who consumed uncooked/raw vegetables were more likely to be infected with intestinal parasites compared to their counterparts (OR = 3.02 ; 95 % CI: 1.64, 14.33; *P* < 0.001) (Fig. 6 C) and (OR = 1.24; 95 % CI: 1.65, 2.37; *P* < 0.001) (Fig. 6 C); respectively.

## Discussion

Women living in low-income countries are at a higher risk of acquiring IPIs that leads to severe anemia. This scenario will put both the mother and the baby at higher risk of morbidity and mortality. This study was conducted to summarize the current evidence of IPIs and associated factors among pregnant women in Ethiopia.

31 eligible studies that have quantified the magnitude of IPIs were included. Accordingly, the pooled prevalence of IPIs among pregnant women in Ethiopia was estimated to be 27.32 %. However, this pooled prevalence is less than studies from Brazil, where 57.1 % pregnant women were harbored at least one parasite [46],Cameroon (31.91 %) [47],India (42.67 %) [48] and Colombia (41 %) [49].

But, our finding is higher than reports from Kenya (24 and 13.8 %) [50, 51], Ghana (23.0 %) [52], Nigeria (20.8 %) [53], Sudan (13.0 %) [54] and other multicounty studies (20 %) [55]. These differences might be attributed to socioeconomic status, poor hygiene and sanitary facilities, weather, climate and environmental factors in the countries. It is also estimated that more than one-third of Sub-Sahara population are infected with at least one species of helminths [56]. Though pregnant women are vulnerable to IPIs, being pregnant was not yet regarded as a significant risk factor for acquiring enteric parasitic infections in Benin [57].

Hookworm and Ascariasis infection were the most prevalent in this review. Similarly, a global systematic review and meta-analysis identified *Hookworm* and *Ascaris lumbricoides* as the leading helminths affecting pregnant women while *Giardia lamblia* and *Entamoeba histolytica* lead protozal infection [16]. The most prevalent types of intestinal parasite identified among pregnant women were *Ascaris lumbricoides* followed and Hookworm in Kenya and Benin [51, 57]. Another study in Kenya identified Hookworm as one of the most common infestation at the first ANC visit [50]. *Giardia lamblia* and *Ascaris lumbricoides* in Colombia [49], *Schistosoma mansoni* and *Trichuris Trichiura* in Ghana [58] were the most prevalent parasitic infections. *Trichuris trichiura* was reported as a predominant parasite followed by *A. lumbricoides* in other study [59] and *A. lumbricoides* was dominantly identified parasites followed by *T. trichiura* Venezuela [60].

In addition, our subgroup analysis indicated that the rates of IPIs among pregnant women varied across different regions of the country, study settings and technique of stool examination. As a result, the prevalence is higher in Oromia and Amhara regional state.

Studies conducted in the community showed a higher prevalence than studies done in institutions with a pooled prevalence of 49.93 % (95 %CI: 20.49, 79.37) and 24.84 % (95 %CI: 19.51, 30.17); respectively. This might be related with inadequate water supply and poor sanitation [61]. On the other hand, studies that used a combination of formalin-ether concentration and direct wet mount stool examination techniques reported a higher prevalence of IPIs (35.99 %; 95 %CI: 26.22, 45.78) than studies that used a single stool examination to diagnose intestinal parasites. This evidenced that the detection rate of intestinal parasites improved by concentrating stool samples [39].

Different factors for IPIs were analyzed and the pooled odds ratio was summarized. As a result, rural residents, being bare footed, poor hand washing habits and eating uncooked/raw vegetables have significant association with the occurrence of IPIs among pregnant women in Ethiopia. Similarly, the high occurrence of parasitic infection has been related to the poor hygiene condition [46] and residence area [16, 47] in other studies. It is also evidenced that factors influencing the continuous transmission of IPIs in sub-Saharan countries include poor sanitation and hygiene and non-availability of potable water for domestic use [53]. In line with our analysis, pregnant women who practiced hand washing regularly had lesser infection in India, Kenya and Benin [48, 51, 57].

Contradicting to this view, the prevalence of intestinal parasites was almost same in both rural and urban pregnant women in India [48]. The finding of this review was consistent with studies in Benin where pregnant women who consume uncooked/raw vegetables from food vendors were more likely to be infected with intestinal parasites. However, in contrary to our review result; being barefooted was not significantly affect the prevalence of IPIs [57]. Consuming unwashed vegetable and being barefooted were associated with IPIs among pregnant women [59].

In line with a review conducted in Sub-Saharan countries [53, 56], we also found that the odds of IPIs were found to be higher in pregnant mothers living in rural areas. Because, place of residence, usually determines people’s lifestyles, income, social and cultural activities, and most notably their health conditions.

### Strengths and limitations

Our meta-analysis tried to elucidate a national figure on prevalence of IPIs during pregnancy. It included studies done both at institution and community settings. Both cros-sectional and case control stud designs were included which enabled us to identify temporal relationship among predictors and outcome variables. As per our search, this is the first analysis done in Ethiopia. However, this meta-analysis is done only on 5 regions of Ethiopia and as a result; the whole image of the problem might be under represented.

## Conclusions

Our systematic review and meta-analysis has estimated a high prevalence of IPIs during pregnancy in Ethiopia. The result indicates the need for priority interventions targeted to improve maternal health during pregnancy. Investing in maternal health is also a key strategy to reduce low birth weight and preterm birth. Apart from socio- economic factors, IPIs during pregnancy are related with poor hygienic practices, being barefooted and eating habits of raw vegetables. Therefore, an optimal personal hygiene and developing shoes wearing habit by the mothers is essential to meet both maternal and child requirements and reduce adverse health consequences in addition to spaced pregnancy. To prevent anemia, pregnant women are advised to take iron, folate supplements, eat iron-rich foods, and prevent intestinal worms.

## Data Availability

The datasets used during the current study are available at the corresponding author upon reasonable request.

## Abbreviations

OR: odds ratio
CI: confidence interval
IPIs: Intestinal parasitic infections
IUGR: Intrautrine growth retardation
STH: soil-transmitted helminths
PC: preventive chemotherapy
PRISMA: preferred reporting items for systematic review and meta analysis
SNNPR: southern national, nationalities and peoples region
ANC: antenatal care
